# Investigation of the Climatic and Environmental Context of Hendra Virus Spillover Events 1994–2010

**DOI:** 10.1371/journal.pone.0028374

**Published:** 2011-12-01

**Authors:** Rosemary McFarlane, Niels Becker, Hume Field

**Affiliations:** 1 National Centre for Epidemiology and Population Research, Australian National University, Canberra, Australian Capital Territory, Australia; 2 Queensland Centre for Emerging Infectious Diseases, Biosecurity Queensland/DEEDI, Brisbane, Queensland, Australia; Veterinary Laboratories Agency, United Kingdom

## Abstract

Hendra virus is a recently emerged bat-borne zoonotic agent with high lethality in horses and humans in Australia. This is a rare disease and the determinants of bat to horse transmission, including the factors that bring these hosts together at critical times, are poorly understood. In this cross-disciplinary study climatic and vegetation primary productivity variables are compared for the dispersed and heterogenic 1994–2010 outbreak sites. The significant occurrence of spillover events within the dry season (p =  0.013, 95% CI (0.57–0.98)) suggests seasonal forcing of transmission across species, or seasonal forcing of virus excretion by the reservoir host. We explore the evidence for both. Preliminary investigations of the spatial determinants of Hendra disease locations are also presented. We find that postal areas in the Australian state of Queensland in which pteropid fruit bat (flying fox) roosts occur are approximately forty times more likely (OR = 40.5, (95% CI (5.16, 317.52)) to be the location of Hendra spillover events. This appears to be independent of density of horses at these locations. We consider issues of scale of host resource use, land use change and limitations of existing data that challenge analysis and limit further conclusive outcomes. This investigation of a broad range of potential climatic and environmental influences provides a good base for future investigations. Further understanding of cross-species Hendra virus transmission requires better understanding of flying fox resource use in the urban-rural landscape.

## Introduction

Hendra virus (HeV) was first identified as a cause of morbidity and mortality in horses and in-contact humans in 1994 [1], [2]. Fourteen spillover events were reported to the end of 2010. These are responsible for the death of 44 horses and 4 humans in Queensland and adjacent northern New South Wales on the east coast of Australia. This is a rare disease but has significant consequences: human and equine case fatality rates are 57% and 75% respectively.

The primary hosts of the disease are flying foxes (black flying fox *Pteropus alecto*, little red flying fox *P. scapulatus*, spectacled flying fox *P.conspicilatus*, and the grey headed flying fox P. *poliocephalus*). The virus is endemic across their geographic ranges, an area collectively larger than that in which disease in horses or humans has been observed. The crude prevalence across all species is 47% [3]. All four species have been affected by natural vegetation clearing and have increased their reliance on vegetation in urban and peri (ex) urban areas [4]. Urbanisation has potentially driven an increased opportunity for pathogen transmission (i.e. spillover) to novel domestic animal and human hosts [5]. It is proposed that urbanisation has also created the opportunity for increasingly isolated sub-populations of flying foxes to have pronounced cycles of waning HeV immunity and reinfection within a meta-population structure [6], [7].

The specific mechanism of bat-horse transmission is unknown, but ingestion by horse of bat excreta, partially eaten food or reproductive fluids are plausible modes [2], [3]. Experimental natural transmission from bats to horses has not been achieved to date [8]. Virus can survive for more than 4 days in flying fox urine at 22°C, but is inactivated in less than 24 hrs in urine or fruit juice at 37°C [9]. Infection in the natural host is not associated with evident clinical disease, and pre-dates infection in horses and humans [3], [10].

Many communicable diseases display pronounced seasonality [11]–[14]. Seasonal changes in host behaviour and contact rates, exposure to pathogens and annual pulses of host, births, deaths and immune status can all affect seasonality of infectious diseases of wildlife [11]. However seasonality of outbreaks of novel disease (i.e. the spillover event) need not mirror seasonality of virus cycling within the reservoir host. Transmission of pathogens *from* wildlife may be seasonally driven as animals follow seasonally available resources. Traditionally flying foxes (with species differences in distribution and non-coastal range) were nomadic over large areas of continuous coastal forest in eastern Australia, utilising local abundances of fruit, pollen and /or nectar [4]. Variation in contemporary resource availability may result in peak times for direct contact between bats and horses. Seasonal and climatic variations may also affect persistence of pathogen in the environment and define the window of time within which indirect contact may occur. HeV is potentially present continuously in the environment of horses in coastal Queensland and northern N.S.W., albeit varying in viral load [7]. Seasonal factors may increase the amount of virus available for ingestion, in line with the current hypotheses of bat – horse transmission [3]. This motivates our primary objective, which is to look for seasonality in the incidence of Hendra spillover events.

The 1994–2010 Hendra spillover event locations are dispersed along 1500km of the eastern coastal plain of Australia. This potentially poses challenges for comparison of spillover sites. An apparent increased frequency of spillover events may at least partially reflect improved surveillance. Alternatively, climatic and land use change may be important underlying drivers for spillover [3], [5], [15]. Quantifying human-induced drivers with their profound effects on wildlife resource availability and redistribution is a major challenge of emerging zoonotic disease research. Spatial, temporal and ecological locational signatures of disease outbreaks have been explored in a number of studies [16]–[24], but not yet within HeV studies.

In this paper we suggest that HeV spillover to horse and associated humans displays a seasonal pattern corresponding to the dry season. We explore drivers of this seasonality including rainfall, temperature, and vegetation primary productivity. As the number of outbreaks is small and the possible drivers of seasonality are many, the exploration of these drivers is considered to be a secondary analysis with the main objective of formulating hypotheses to be investigated in future. Rainfall and temperature are examined as determinants of pathogen persistence in the environment and primary productivity as a proxy for horse and bat resource availability. Evidence for seasonal reservoir host (flying fox) reproduction and movement patterns and seasonal amplification host (horse) factors are discussed. We also explore long-term trends in rainfall, temperature and vegetation primary productivity at disease sites as an insight to climate and land use change driving of local disease emergence. Host distribution and density is described for the single year of concurrent data availability. Our preliminary studies provide a starting point to understand the critical environmental factors which bring reservoir hosts and novel hosts together to enable HeV transmission to occur. Ultimately, such understanding will enable strategic prevention and risk minimization activities, and aid in the management of conflict between flying foxes and humans.

## Results

### Evidence for seasonality

**Figure 1:**
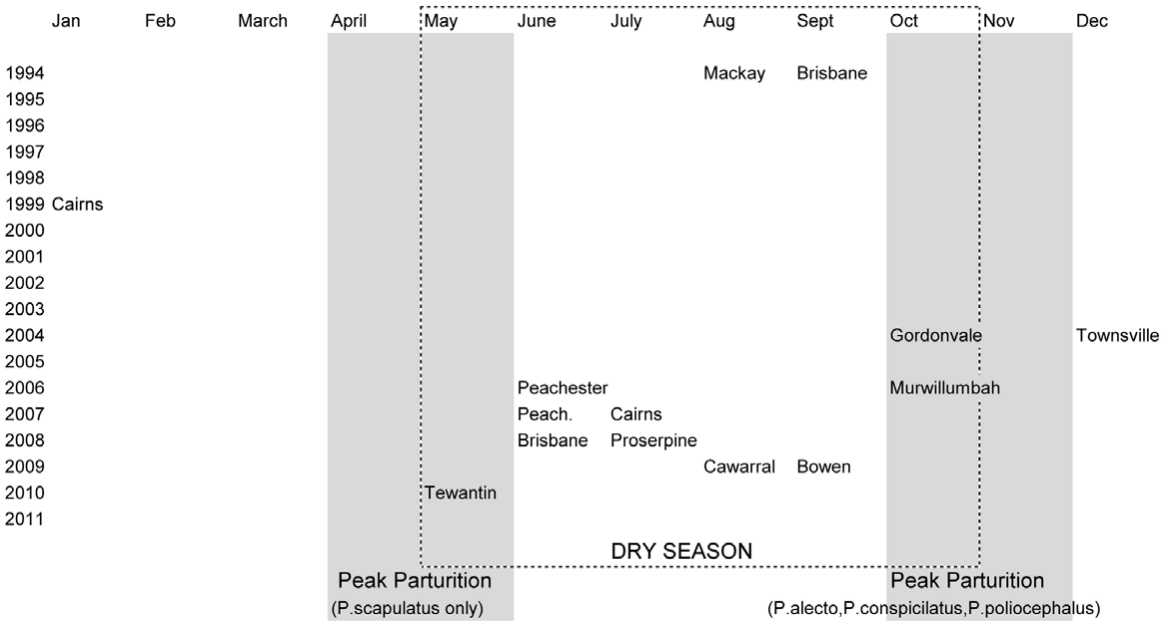
Distribution of Hendra spillover events 1994–2010 (n = 14 locations) by month and year. Also shown are dry season and peak parturition months for little red flying fox *Pteropus scapulatus* (Apr/May) and black flying fox *P. alecto*, spectacled flying fox *P.conspicilatus*, and the grey headed flying fox *P. poliocephalus* (Oct/Nov).

From 1994–2010, 12/14 Hendra disease events have occurred in the dry season (May–October in the southern hemisphere tropics) (Figure 1), providing statistically significant evidence that π, the probability that a spillover event occurs in a dry season, is not 0.5 (p = 0.013, two tailed test). The 95% confidence interval for π is 0.57–0.98.

**Figure 2:**
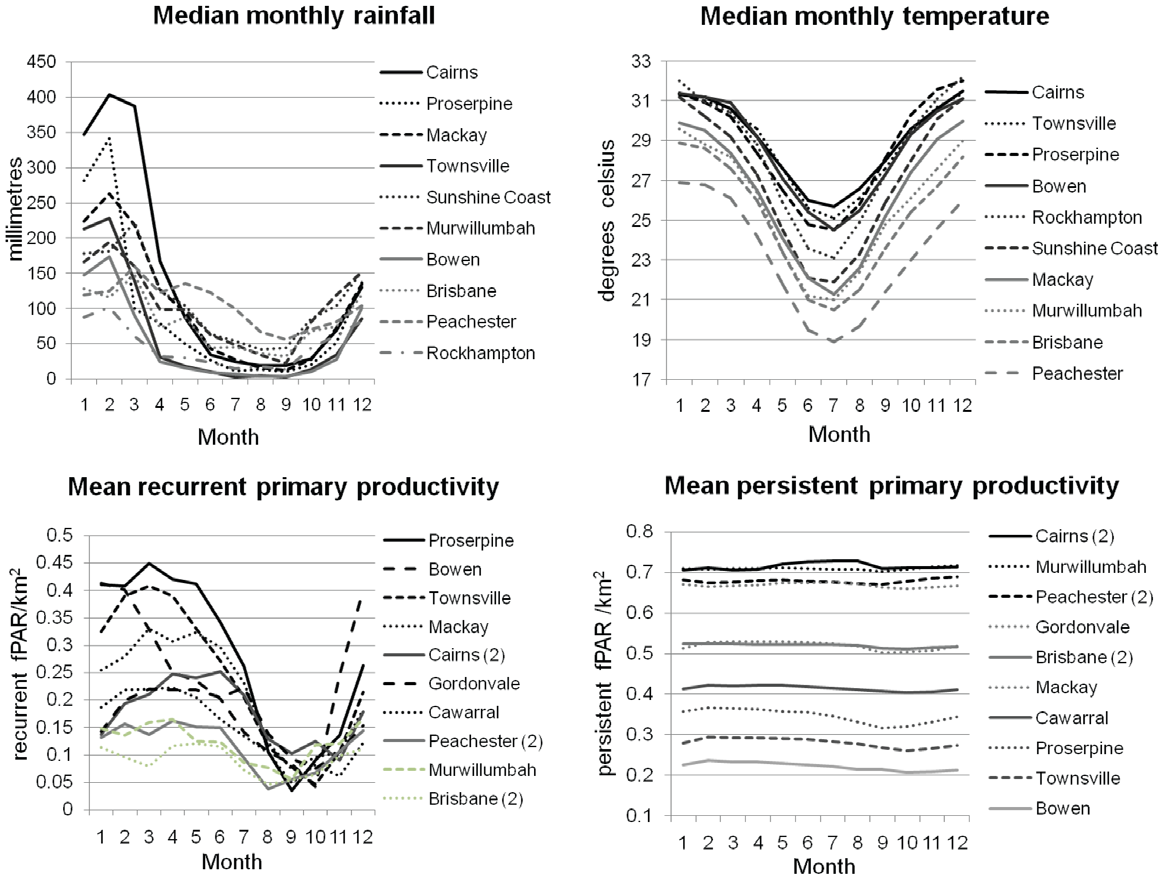
Monthly rainfall, temperature, recurrent and persistent vegetation primary productivity at Hendra spillover event sites, 1994–2010. Median monthly rainfall and temperature as recorded at the 10 closest meteorological stations to the 14 spillover events (see text for details); and mean recurrent and persistent vegetation primary productivity at Hendra disease event locations 1–13 extracted from AVHRR 1km pixel data, 1995–2006.

Figure 2 illustrates that May to October are the months of lowest temperature and rainfall for all spillover event sites. A corresponding decline in recurrent primary productivity, predominantly representing herbage, is apparent but occurs 1–2 months later. Mean persistent primary productivity displays variable seasonal patterns, peaking in the dry season only in more tropical locations.

### Within-season variation in rainfall, temperature and vegetation primary productivity

Temperature, rainfall and primary productivity differ greatly at the time and location of each spillover event and were not extreme. Consequently we did not seek comparison between locations. However, no significant associations were demonstrated between event-month and within-season variation in rainfall, temperature or primary productivity at each event location. For months in which spillover events occurred, the range of mean monthly temperatures was 17.7°C–31.8°C, rainfall: 0.2–273 mm, and days without rain: 13–30 days. Eight sites had days ≤22°C within the month of spillover event. When measured at 1 km pixel resolution, the difference in the mean persistent (tree canopy) primary productivity at Hendra spillover sites in the year of event compared to the mean in the preceding 4 years (for each site) approached positive statistical significance (p  =  0.077, two sided test. The 95% CI for the difference in means was 0.36–3.14. In contrast, the difference in means did not approach significance when using 8 km pixel or 250 m pixel data.

### Examination of seasonally varying host factors

The temporal pattern of Hendra events corresponds to a period 6–9 month following peak parturition of the black, grey-headed and spectacled flying foxes (see Figure 1). This is also when mating occurs in these species and parturition occurs in little red flying foxes [4].

### Possible environmental drivers for the local emergence of Hendra

The observed increase in frequency of Hendra spillover events (11/14 events from 2004–2010) is not obviously associated with a climatic trend: half of events were less than or equal to the median for each site (and to the median for the Climate Reference Normal decades 1960–1990) for rainfall in year of event, month of event and temperature in month of event. The positive association with El Nino events (OR = 1.66, 95% CI (0.55, 5.01)) was not statistically significant at the 0.05 level. Districts in which Hendra events occurred were drought declared (n = 3), not drought declared (n = 9), or not covered by map archive (1994, n = 2). There were no drought declared districts in Queensland in 1991, 1999 or 2011. Between 2002 and 2010, up to 65% of the land area of the state, including much of S. E. Queensland, was drought declared.

Long-term primary productivity trends at incident sites (AVHRR data 8 km pixel) for persistent fPAR between 1981–2006 were increasing (7), decreasing (5), and for recurrent fPAR increasing (5) or decreasing (7). Land-use at these sites was classified (1999–2002) urban residential (3) rural residential (7), grazing natural vegetation (2) and irrigated seasonal vegetables and sugar (2). All sites were associated with remnant native vegetation managed for conservation or grazing within 1 km of incident sites. Current (1995) vegetation at and within 1 km of incident co-ordinates was listed as unknown (i.e. non-native) as the primary vegetation type for 12 sites: the remainder, and the secondary vegetation of half of all sites, reflected pre-European vegetation types. Pre-European (1750) vegetation was primarily tall eucalypt forest (9), eucalypt woodland (1), melaleuca forest and woodlands (2), rainforest and vine thickets (1), eucalypt woodland with tussock grassland (1). We observe that these parameters vary substantially across the east coast of Queensland and N.S.W. We do not report statistical analyses or present denominator values as the relevant spatial scale is yet to be developed and existing data are insufficient for long term comparison within-sites. The National Vegetation Information System provides an overview of deforestation and vegetation change [25]. Regions previously dominated by eucalypt- and rain- forest approximate the current distribution of flying fox roosts and Hendra events in Queensland and north N.S.W.

**Figure 3:**
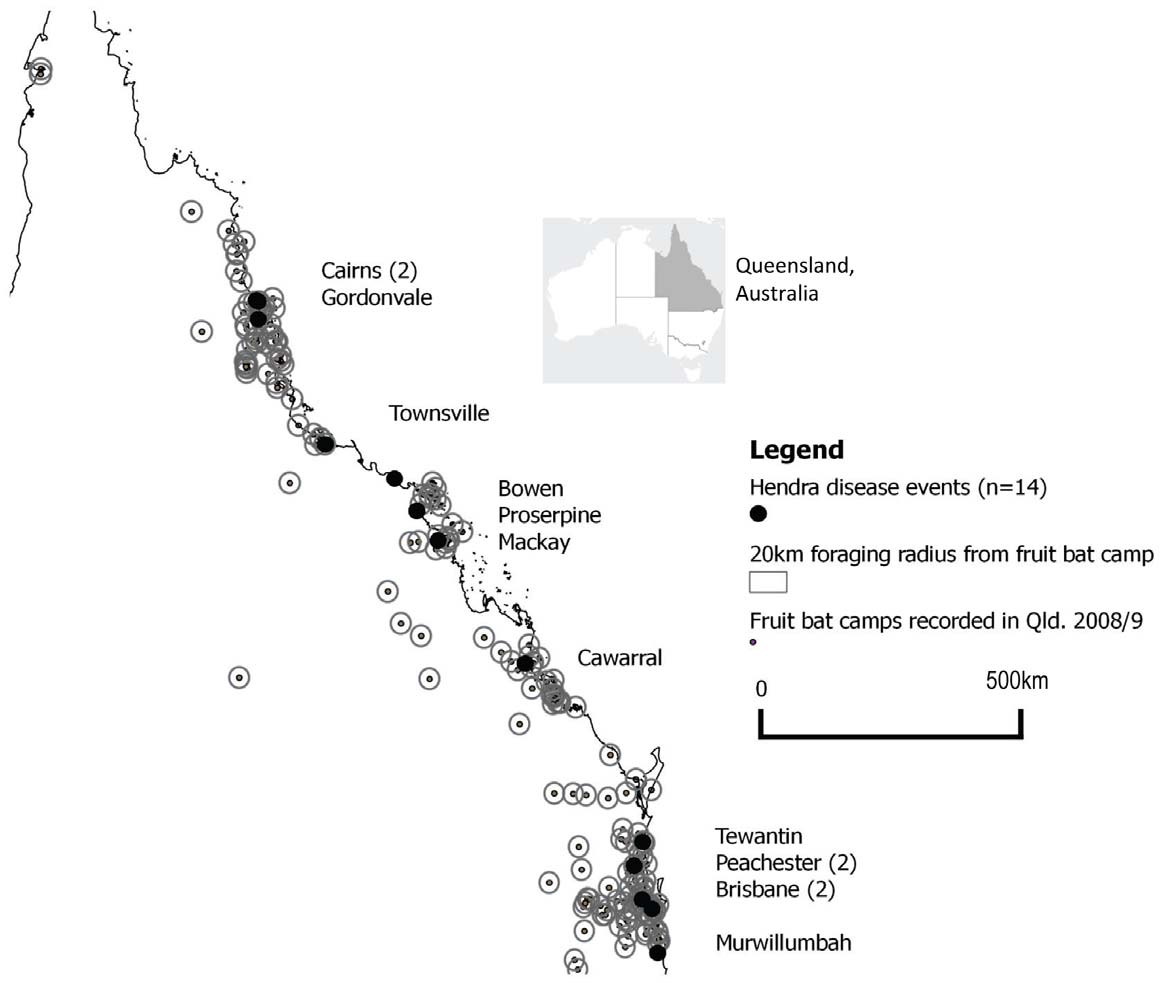
Recorded flying fox roosts, Queensland (2008/9) and location of Hendra spillover events (n = 14), 1994–2010.

**Figure 4:**
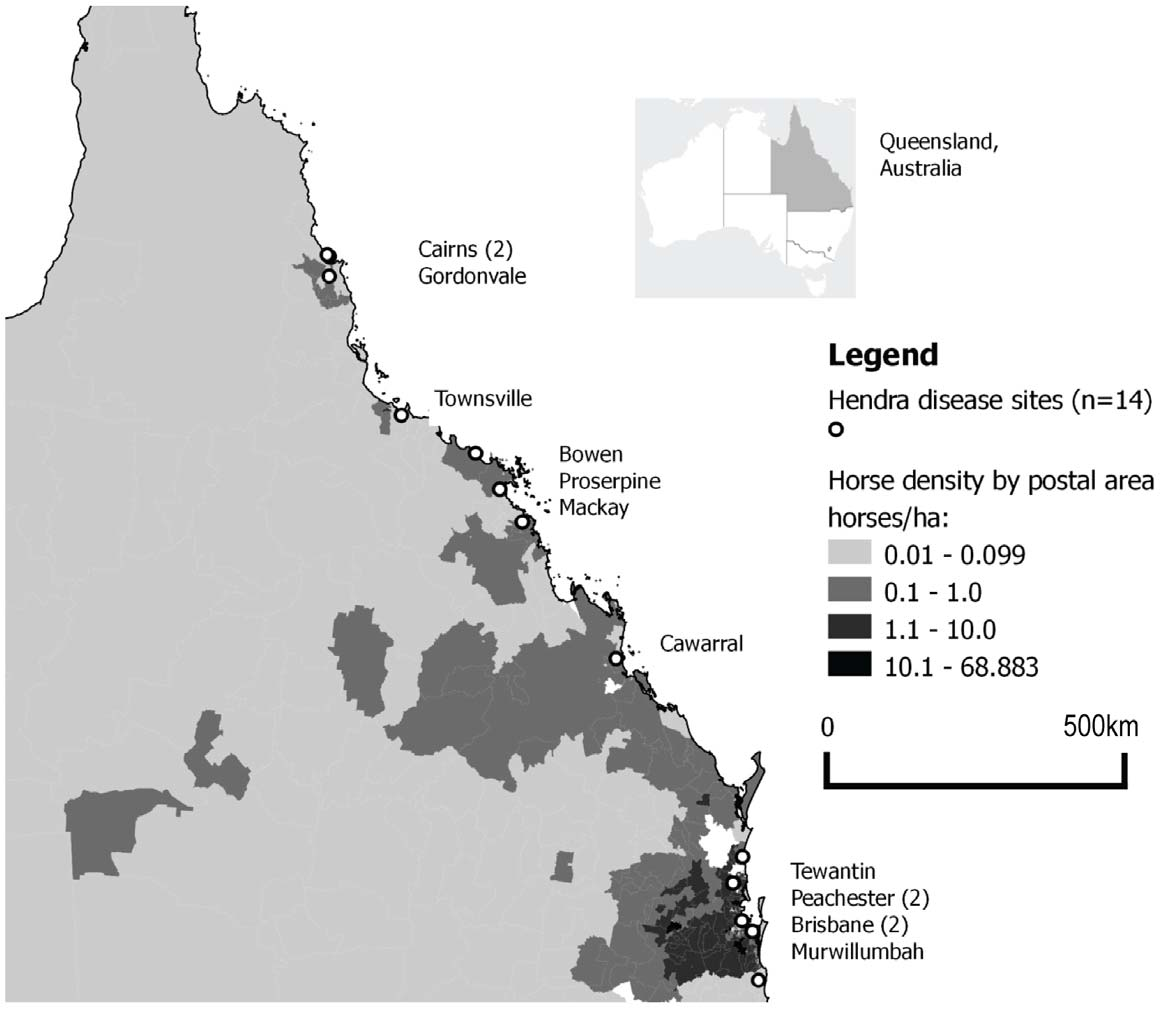
Horse density by postal area, Queensland (2007/8) and location of Hendra spillover events (n = 14), 1994–2010.

Hendra spillover events have occurred within the east coast distribution of the black flying fox and little red flying fox. This overlaps with the grey headed flying fox to the south and spectacled flying fox to the north. Spillover locations are within a 20 km radius (an average range of nightly feeding activity [4]) of 2–11 (mean 4.6) recorded flying fox roosts, except at Bowen where no roosts are mapped within 50 km of the spillover site (see Figure 3). Ten events occurred within 20 km of a permanent roost. Equine density calculated by postal area was greatest in south eastern Queensland (centred on the state capital, Brisbane) where densities were in the order of 11–70 horses/ha. This is also where human population density is greatest (6.6–53.9 people/ha in 2006). Increasing human population (1991–2006) of varying magnitude occurred at all spillover sites except one (Bowen) which had a stable population and one (Gordonvale) where population was decreasing. Horse density was locally increased around major towns along the coast and these ranged between 1–10 horses/ha (see Figure 4). However 2/14 events occurred where horse density was<0.1 horse/ha. Only 4 events have occurred at commercial multi-horse enterprises, other affected horses were individual recreational horses. An odds ratio of 40.5 (95% CI (5.16, 317.52) p<0.0001) is calculated for the odds that Hendra disease will occur in a postal area containing flying fox roosts. However Hendra events were not significantly associated with postal area having horse density ≥0.1 horse/ha independently or where flying foxes are also present.

## Discussion

This study demonstrates a significant association with the dry season for spillover events 1994–2010. A consistent pattern of lower rainfall, temperature and recurrent primary productivity is evident at all sites between May and October. However, no pattern of within-seasonal variation emerges that distinguishes event months from non-events. Conditions for environmental persistence of active virus are not met more frequently in the month of events. Lower temperatures occur regularly for 1–2 months mid-year in the southern locations where the majority of events occur (6 events have occurred in the S.E. Queensland bioregion, which includes the northern N.S.W. event). Peachester, where two spillover events have occurred, has 5 months with median monthly temperatures below 22°C annually (and is also closest to a permanent flying fox roost of all locations). It is extremely unusual for temperatures of 37°C, or greater, to occur at any of these coastal sites. No other pattern was discerned from within-season variation of rainfall or temperature.

Hendra event sites vary significantly in latitude, climate, biogeography (including vegetation communities, deforestation, flying fox species composition), human and equine populations. In the face of this heterogeneity we have sought evidence of within-location variation that may indicate a climatic and ecological signal associated with spillover events. Significant associations with vegetation primary productivity were not demonstrated. Vegetation resources are difficult to summarise over large areas due to the diversity of plants used by the four flying fox species of concern [4]. However, flying fox movements are directed to abundance of preferred food [4] and primary production provides a consistent long term measure of plant photosynthetic activity that reflects such variation [26]. Annual and inter-annual production of nectar, blossom, fruit and foliage vary for different trees and shrubs not only spatially and with season but with important climatic effects such as ENSO [4]. The weak positive association demonstrated between spillover events and regional El Nino periods is not reflected in the more site specific examination of variables. Primary productivity within the urban-rural land use zones (in contrast to natural areas) is likely to be less predictive of wildlife activity due to other influences of urbanisation [27]. However, Hendra spillover events have occurred in proximity to recorded flying fox roosts and so this study was designed to explore these parameters at a local scale. We do not find evidence that spillover sites are significantly more (or less) productive at the time that spillover occurs than that month, season or year compared for the previous five years, or within the previous 6 months.

The variation in primary productivity at spillover events, and over the longer intervals examined, is at least in part due to land use and vegetation diversity and change at these locations but selecting the appropriate scale of resolution at which to look for patterns is problematical. A restricted time frame (5 years) was chosen to limit changes in land use, horse occupancy, tree resource maturation, viral cycles etc. likely to change significantly over longer time frames within sites. We analysed primary productivity data without transformation or aggregation as the most appropriate scale for analysis is unknown. In these landscapes a 1 km pixel captures an entire farm (in most cases) and approximates the accuracy of disease locations. An 8 km pixel may accommodate the majority of flying fox foraging activity in urban-peri urban areas [28]. The importance of individual trees cannot be captured within these data and yet are known to influence flying fox movements [28]. The magnitude of primary productivity at the disease event site relative to the entire foraging range of local flying foxes, or regionally (to account for hypothesised between-population reintroduction of virus) was not investigated and requires further exploration.

This study does not demonstrate a strong climatic or ecological signal at a disease location level. In studies of Ebola virus spillover events in central Africa, all outbreaks occurred within a signature vegetation class following sharply drier conditions at the end of the rainy season [24]. The strong associations in the Ebola studies (at AVHRR data 8 km pixel resolution) have enabled more sophisticated analyses to be undertaken [21], [29]. No correlation with rainfall, temperature, quantity of food, parturition period or roost was observed in a study of Nipah virus excretion by Lyle’s flying fox (*Pteropus lylei)* in Thailand [30]. However a distinct seasonal pulse in shedding was observed (in May) that corresponded with the dispersal of juveniles.

An increased cycling of virus in black, grey and spectacled flying fox pups at around 6 months of age, an age at which many mammals loose maternal antibody protection [31] would overlap the observed dry season pattern of incidence. Social contact associated with mating in these species needs also to be considered as a source of increased virus shedding [31]. However parturition, which occurs in little red flying foxes at this time, has been shown to be associated with increased Hendra seroprevalence [32]. The asynchronous breeding cycle of little red flying foxes (and/or year–round breeding of black flying foxes in the north of their range [4]) could account for susceptible pups shedding virus to horses in the two wet season events. The relative magnitude, and hence importance, of these potential sources of seasonal virus activity have not been quantified. The magnitude and duration of effect of maternal antibodies to Nipah virus in Lyle’s flying fox pups in Thailand is also unknown [30]. Regularly observed migrations of the nomadic little red flying fox into coastal Queensland, hypothesised to periodically reintroduce infection to the coastal species, do not reflect the timing of the observed distribution of spillover events to horses [3], [4].

Increased transmission influenced by seasonal horse management, behavior or susceptibility is difficult to examine. It may be that there is an increase in interest for horses in peri-urban paddocks to consume potentially virus-contaminated under-tree grass, foliage or partially eaten fruit during the cool, dry, low grass producing months. Avoiding grazing under trees and ensuring feed bins are covered and not placed under trees is now standard risk minimization advice [33]. Data on property level risk management prior to spillover events are not available for analysis. The majority of the affected sites have involved non-commercial grazing of non-breeding, mature, mares [6]. Our preliminary spatial analyses suggest that horses within the foraging range of coastal flying fox roosts are at increased risk of Hendra disease, irrespective of horse density. The association between disease event sites, predominantly recreational horses and adjacent native vegetation warrants further consideration.

### Conclusion

A significant occurrence of Hendra spillover events 1994–2010 within the dry season is demonstrated. We do not find within-season variation that distinguishes events from non-events. Minimum climatic conditions for persistence of virus in the environment are met at all disease locations throughout the year with varying seasonal amplification. Location-specific evidence for climatic and environmental drivers of Hendra disease is limited. It is unclear at this stage whether these drivers may be important at the scale of infected property, local flying fox population or meta-population level. The majority of spillovers have occurred within the nightly foraging range of recorded flying fox camps, in urban, peri-urban and rural locations within a range of horse (and human) population densities. We consider host factors such as waning immunity in pups and parturition in little red flying foxes to be a possible mechanism for seasonal forcing of virus transmission from local bats. It is likely that some combination of these environmental and host factors exists, reflecting the epidemiological concept of necessary and sufficient cause. A rigorous yet robust approach is required to examine climatic and ecological variables where data is sparse and many unknowns exist. The methodology presented here may be useful in other studies of emerging infectious diseases of wildlife origin.

## Materials and Methods

The location and occurrence of Hendra spillover events were identified on the basis of documented, clinically described, and laboratory-confirmed cases. Spillover event sites are defined as the location at which the index (equine) case of infection was first identified as unwell and subsequently diagnosed. Month of spillover event is the unit of time employed. The precise day of bat-horse transmission for each event is unknown; experimental and field observations suggest an incubation period of 5–16 days for horses [8], though incubation periods up to 31 days have been considered.

### Evidence for seasonality

The sign test is used to look for evidence that dry season (May–October) is a predictor for Hendra spillover events. This wet-dry seasonal effect decreases with increasing latitude so we investigate the persistence of this climatic pattern across the study area, and the corresponding vegetation response. The median monthly rainfall and temperatures recorded at Australian Bureau of Meteorology stations (www.bom.gov.au) within 25 km of the 14 spillover locations (stations: 033119; 40237; 40265; 31011; 032040; 040121; 58158; 33247; 39083; 33096; 33257; 40059; 40908) were used for analysis.

Monthly fPAR (fraction of Photosynthetically Active Radiation absorbed by vegetation) derived from Advanced Very High Resolution Radiometer reflectances (AVHRR) were used as the measure of vegetation primary productivity at disease sites. These data describe the monthly fraction of fPAR as grids of 0.08 degree cell size from July 1981 onwards and of 0.01 degree from February 1995 to December 2006. In these data fPAR is split into that of persistent vegetation and of recurrent vegetation, which represent non-deciduous perennial vegetation and annual, ephemeral and deciduous vegetation, respectively [26]. The Moderate Resolution Imaging Spectroradiometer (MODIS) dataset of fPAR at 250 m resolution split into persistent and recurrent vegetation (courtesy S. Berry, [34]) for the period from January 2001 to June 2007 was also examined. Missing data were interpolated with weighted averages. We analysed fPAR data from disease sites at the raw resolutions available: AVHRR 8 km pixel (events 1–7), AVHRR 1 km pixel (events 3–6); MODIS 250 m pixel (events 4–8). Mean persistent and recurrent fPAR at 1 km resolution was calculated for the disease outbreak locations for the decade 1995–2006.

Seasonal patterns of rainfall, temperature and primary productivity at these locations are examined graphically. These parameters are also examined for the 14 spillover events.

### Within-season variation in rainfall, temperature and vegetation primary productivity

We examined whether the value of rainfall, temperature and primary productivity of vegetation in the month of a spillover event was significantly different to non-event months, using t-tests and a distribution-free method based on ranks. A restricted time frame (5 years) was chosen to limit the other uncontrolled variables. The variable of interest (monthly rainfall, temperature, persistent and recurrent fPAR) for the preceding 12 months, the current dry season and month of spillover event was ranked against the corresponding interval for the previous 4 years. This was repeated for the month of event and the preceding 3 and 6 months. We also compared these variables in the month of event to the mean of non events in the same sets of time intervals. Days without rain were compared for month of event with the same month in the preceding four years and preceding 6 months. Post-event years were not included in analyses as it was considered likely that risk minimising behavior post-incident would mask any further expression of disease.

For the comparison based on ranks, the rank of event scores relative to local non-event scores was determined, as was the rank expected under the assumption of no effect. The rank of event scores and their expectations were accumulated over the event sites and compared using a two-tailed z-test.

### Examination of seasonally varying host factors

Timing of annual pulses of births, predictable seasonal migration patterns, and loss of maternal antibody protection of fruit bats was derived from the literature. Those concurrent with disease events are described.

### Possible environmental drivers for the local emergence of Hendra

Rainfall and temperature in the month of spillover event (n = 14) were compared to the median for all years and to the Climate Reference Normal (1960–1990). Climatic data were available for 20–80 years from the selected stations listed above**.** Occurrence and duration of El Nino/Southern Oscillation (ENSO) periods in Queensland and northern N.S.W. were extracted for 1990–2010 (http://www.bom.gov.au/climate/enso/enlist/index.shtml). The odds ratio of Hendra spillover events occurring in months when ENSO effects were experienced was calculated using epidemiological software [35]. The drought declaration status of districts at the time of spillover events was also recalled from state authorities (http://www.longpaddock.qld.gov.au/queenslanddroughtmonitor/queenslanddroughtreport and http://www.dpi.nsw.gov.au/agriculture/emergency/drought/situation/drought-maps/drought-maps).

A geospatial information system was developed (Quantum GIS) to compare the attributes of spillover event sites, and to extract and aid in the interpretation of primary productivity data (ArcView 9). Vegetation composition at event sites was examined using the Australian National Vegetation Information System (current (1995) and pre-European (1750) vegetation (http://www.environment.gov.au/erin/nvis/index.html). Land use at the event sites was assessed from Queensland Land Use Mapping Program (1999) (http://www.derm.qld.gov.au/science/lump) and Land Use of Australia, Version 3–2001/2002 (http://adl.brs.gov.au/anrdl/metadata_files/pa_luav3r9eg__00112a06.xml).

Information on host distribution and density was sought to elucidate critical host factors. The best data for horse and flying fox roost distribution are available concurrently for a single year and were used to provide a one point in time snapshot of host distributions. Roost locations (2008/9) were obtained under license from Queensland Department of Environment and Resource Management and New South Wales Department of Environment and Climate Change. Horse population location data collected systematically in Queensland and N.S.W. 2007/2008 were obtained under agreement from Biosecurity Queensland and Department of Agriculture Fisheries and Forestry. Human population data was obtained from census data (1991, 1996, 2001, 2006) under licence from Australian Bureau of Statistics. This was aggregated at postal area level to compare densities and to calculate odds of disease. Postal areas are Australian Bureau of Statistics mappable approximations of Australian postcodes and form the smallest unit for processing census data. Proximity of event sites to flying fox roosts, their numbers, permanence and species composition as recorded were calculated.

#### Addendum

Multiple Hendra spillover events have occurred in 2011: at time of submission 17 events have occurred in June, July and August and one in October, giving further support to pursuing the source of (dry) seasonal forcing of HeV disease.
